# Relationship between High-Performance Liquid Chromatography Fingerprints and Uric Acid-Lowering Activities of *Cichorium intybus* L.

**DOI:** 10.3390/molecules20059455

**Published:** 2015-05-22

**Authors:** Chun-Sheng Zhu, Bing Zhang, Zhi-Jian Lin, Xue-Jie Wang, Yue Zhou, Xiao-Xia Sun, Ming-Liang Xiao

**Affiliations:** School of Chinese Pharmacy, Beijing University of Chinese Medicine, Beijing 100102, China; E-Mails: 20130931518@bucm.edu.cn (C.-S.Z.); wangxuejie09@sina.com (X.-J.W.); Zhouyuewolf@163.com (Y.Z.); Sunxiaoxia321@sina.com (X.-X.S.); Shirley0601211@126.com (M.-L.X.)

**Keywords:** chicory, spectrum-effect relationships, uric acid-lowering

## Abstract

This study aimed to explore the spectrum-effect relationships between high-performance liquid chromatography fingerprints and the uric acid-lowering activities of chicory. Chemical fingerprints of chicory samples from ten different sources were determined by high-performance liquid chromatography, and then investigated by similarity analysis and hierarchical clustering analysis. Pharmacodynamics experiments were conducted in animals to obtain the uric acid-lowering activity information of each chicory sample. The spectrum-effect relationships between chemical fingerprints and the uric acid-lowering activities of chicory were established by canonical correlation analysis. The structures of potential effective peaks were identified by liquid chromatography with tandem mass spectrometry. The results showed that a close correlation existed between the spectrum and effect of chicory. Aesculin, chlorogenic acid, chicoric acid, isochlorogenic acid A/B/C and 13,14-*seco*-stigma5(6),14(15)-diene-3α-ol might be the main effective constituents. This work provides a general model of the combination of high-performance liquid chromatography and uric acid-lowering activities to study the spectrum-effect relationships of chicory, which can be used to discover the principle components responsible for the bioactivity.

## 1. Introduction

*Cichorium intybus* L., commonly known as chicory, is a perennial herb of the Asteraceae family. In the last years, there has been a growing interest in chicory due to its broad pharmacological action, including antibacterial, anti-inflammatory, anti-oxidant, antidiabetic, hepatoprotective, antitumor, anti-hyperlipidemic, hypoglycemic effects and so on [1,2,3,4,5]. Chicory contains a number of medicinally important phytoconstituents, mainly belonging to the alkaloid, phenolic acid, sesquiterpene lactone, aliphatic compounds and their derivatives, volatile oil, flavonoid, and polysaccharide classes, *etc*. [6,7]. Historically, chicory was grown by the ancient Egyptians as a medicinal plant, vegetable, and forage plant, *etc*. [6]. Nowadays, its leaves and roots are still often used for making salads and vegetable dishes, while the roots can also be processed and used as a coffee substitute or food ingredient. According to the FDA, chicory extract fits the category of “generally regarded as safe” (GRAS) and appears in the list of Everything Added to Food in the United States (EAFUS) [8]. In addition, some studies have proved the health benefits of chicory when used as a food or medicinal plant [9]. For instance, in India, the whorls are made into a decoction and used for the treatment of liver disorders, gout and rheumatism [6].

Uric acid (UA) is the end product of nucleic acid metabolism. When the blood UA levels exceed the normal reference interval the resulting condition is generally defined as hyperuricemia. A number of epidemiological reports have revealed that hyperuricemia is not only the central biochemical cause of gout, but also a precursor of cardiovascular diseases, including hypertension, coronary artery disease, cerebrovascular disease and vascular dementia [10,11]. High blood uric acid levels also have a close relationship with kidney disease and metabolic syndrome [11]. In our previous research, quail was used to establish a hyperuricemia model, because its UA metabolic process is similar to humans’, that is, both quail and humans cannot oxidize UA into the more soluble compound allantoin due to their lack of the enzyme uricase [12,13,14]. In previous studies, we found chicory could reduce serum UA levels, inhibit liver xanthine dehydrogenase and xanthine oxidase in quail [15]. However, to the best of our knowledge, little information on the systematic quality evaluation of chicory, and the major effective components in the UA-lowering actions remain unclear so far.

As an important analytical method, high-performance liquid chromatography (HPLC) has been widely applied to assess the quality of herbal medicines for its convenience to operate, fully automatable technique with high resolution, selectivity, sensitivity as well as accuracy [16]. Liquid chromatography with tandem mass spectrometry (LC/MS^n^) detection provides useful structural information, distinguishes compounds with identical molecular weights, and allows for tentative compound identification when standard reference compounds are unavailable. All these characteristics make LC/MS^n^ a powerful tool for mapping the chemical profiling of herbal medicines [17]. Therefore, in this study, HPLC is applied to establish the fingerprints of chicory samples from different sources and collection times. Serum UA is selected to evaluate the therapeutic effects of chicory in the treatment of hyperuricemia. By the combination of HPLC fingerprints with UA-lowering activities, the spectrum-effect relationships of chicory were investigated to screen the effective components with UA-lowering activities. The chemical structures of the screened effective components were determined by LC/MS^n^. This study aims to reveal the effective components of chicory for the treatment of hyperuricemia and quality control of chicory, and provide a useful model for screening effective components from herbal medicine as well.

## 2. Results and Discussion 

### 2.1. HPLC Experiment Results

#### 2.1.1. HPLC Experiments

**Figure 1:**
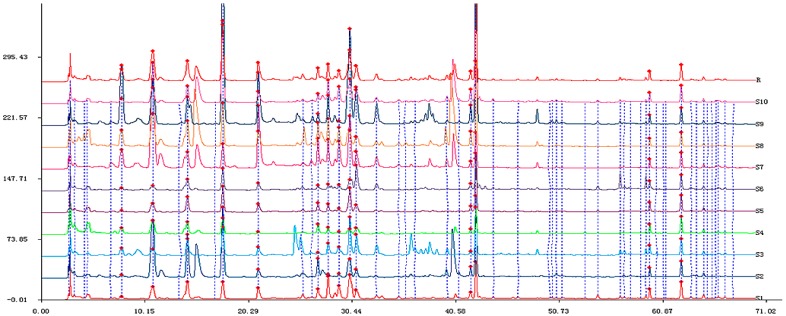
The HPLC fingerprints of the extracts of various chicory and reference standard fingerprint from the 10 chromatograms.

**Figure 2:**
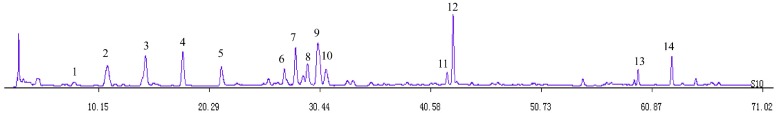
The reference atlas from the 10 chromatograms of chicory.

The methodology validation results showed that the precision of the same sample solution was in the range of 0.02%–0.13% for retention times (t_R_) and 0.87%–4.36% for peak areas of common peaks. The repeatability of this experiment was in the range of 0.02%–0.23% for t_R_ and 0.85%–4.83% for peak areas of common peaks. The sample stability was below 0.21% for t_R_ and 5% for peak areas of common peaks. All results indicated that the developed HPLC fingerprint method was valid and suitable for the sample analysis. The optimized HPLC fingerprints of ten chicory samples and the reference standard fingerprint are shown in Figure 1. Fourteen peaks with large areas and good segregations were selected as the ‘common peaks’ (Figure 2). Peak 2 from S2 was defined as the reference peak to calculate the relative peak areas of other common peaks. Table 1 shows the relative average peak area and t_R_ of fourteen common characteristic peaks.

#### 2.1.2. Similarity of Fingerprints

The similarities between the reference standard fingerprint and the chromatographic fingerprints from ten batches of chicory samples were compared, and their similarity values were 0.89, 0.92, 0.92, 0.92, 0.80, 0.80, 0.93, 0.85, 0.80 and 0.76, respectively. The differences of correlation coefficients further showed variation of the fingerprints and internal qualities of these samples.

#### 2.1.3. Results of HCA

**Figure 3:**
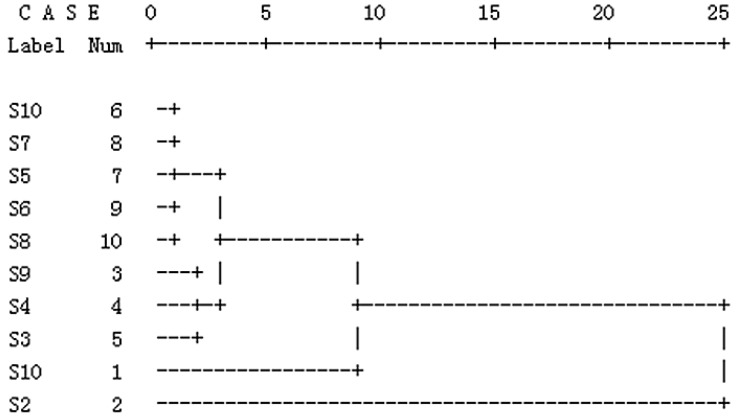
Hierarchical clustering analysis of chicory samples.

HCA is a well-known method for discriminating different samples which has been widely used in classification according to the common peaks. The results of HCA in Figure 3 clearly show that the samples could be divided into three clusters. Cluster I consisted of sample 2, which was collected from Haidian, Beijing. Cluster II included sample 10, which was purchased from Changji, Xinjiang. Cluster III included sample 1, 3, 4, 5, 6, 7, 8 and sample 9, which were respectively collected or purchased from Dalian, Liaoning; Dengta, Liaoning; Zhaoqing, Heilongjiang; Moyu, Xinjiang; Neimenggu; Pingshan, Hebei; Shouguang, Shandong; Changji, Xinjiang . The results may be attributed to different conditions of climate, soil and light, *etc*. For example, sample 2 (from Haidian, Beijing) and sample 8 (from Shouguang, Shandong) were divided into different clusters, this means that the origin might affect the quality of chicory.

**Table 1:** The average retention time and relative peak area of every common characteristic peak.

Peak NO	Retention Time	Average Relative Peak Area of Every Common Peak									
		S1 ^a^	S2 ^a^	S3 ^a^	S4 ^a^	S5 ^a^	S6 ^a^	S7 ^a^	S8 ^a^	S9 ^a^	S10 ^a^
1	7.8	0.05	1.20	0.10	0.06	0.16	0.11	0.02	0.03	0.39	0.37
2	10.9	0.26	1.00	0.33	1.23	0.16	0.27	0.18	0.10	0.75	2.30
3	14.3	0.33	0.27	0.30	0.66	0.27	0.23	0.24	0.22	0.52	0.32
4	17.7	0.25	3.15	1.31	1.16	0.38	0.23	0.33	0.30	0.62	0.50
5	21.1	0.18	0.88	0.18	0.13	0.13	0.03	0.12	0.11	0.27	1.09
6	27.1	0.14	0.14	0.11	0.23	0.04	0.13	0.11	0.08	0.27	0.32
7	28.1	0.27	0.34	0.27	0.05	0.15	0.09	0.10	0.07	0.37	0.35
8	28.7	0.09	0.11	0.07	0.02	0.14	0.01	0.03	0.04	0.10	0.10
9	30.3	0.50	1.72	0.52	0.41	0.58	0.10	0.33	0.26	0.21	0.81
10	30.8	0.17	0.59	0.22	0.13	0.33	0.12	0.12	0.40	0.22	0.24
11	42.0	0.07	0.18	0.15	0.11	0.04	0.04	0.05	0.06	0.15	0.07
12	42.5	0.36	2.67	1.33	0.86	0.54	0.27	0.48	0.34	0.83	0.34
13	59.6	0.07	0.07	0.07	0.07	0.07	0.06	0.06	0.08	0.05	0.06
14	62.6	0.15	0.15	0.15	0.14	0.15	0.15	0.15	0.13	0.13	0.14

a: Average of three experiments.

### 2.2. Results of UA-Lowering Experiments

**Figure 4:**
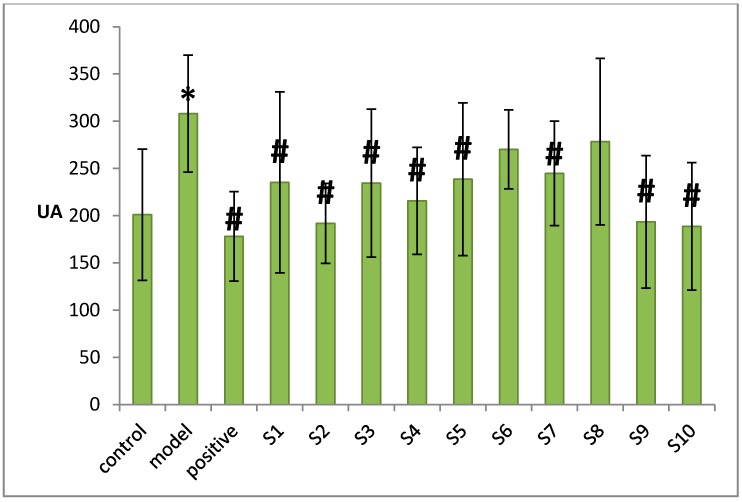
Results of UA-lowering activities. The rates of UA-lowering for 13 groups were presented as mean ± SD (*n* = 10) and the data were statistically evaluated by one-way ANOVA. *p <* 0.05 was considered statistically significant (compared with the control group * *p* < 0.05, compared with the model group ^#^ *p* < 0.05).

The statistical serum UA level results suggested that the model group had a significant higher level compared with control group (*p <* 0.05), which indicated that the hyperuricemia model was built successfully. Both the therapeutic groups (the groups respectively given 10 batches of chicory) and the positive group had statistically significant differences (*p <* 0.05) compared with the model group, except the groups given samples 6 and 8. Figure 4 showed that the magnitude of UA-lowering capability of 10 batches of chicory from max to min is sample 10, 2, 9, 4, 3, 1, 5, 7, 6 and sample 8. Sample 10 exhibited the highest UA-lowering capacity, on the contrary, sample 8 demonstrated the weakest. Among these chicory, sample 10, 2 and 9 showed stronger UA-lowering activities than others, while the effects of sample 6 and 8 lower than other groups, which might be attributed to the contents of effective components.

### 2.3. Spectrum-Effect Relationships between HPLC Fingerprints and UA-Lowering Activities

In this paper, canonical correlation analysis was employed to deal with the spectrum-effect relationships of chicory, which has been proved to be simple and operative. The canonical correlation analysis between UA-lowering activities and peak areas of fourteen common characteristic peaks in the HPLC fingerprints was achieved using the SPSS 17.0 statistical software. The results showed that peak 1, 2, 5, 6, 7 and 11 in HPLC fingerprints possess a close correlation on the UA-lowering activities of chicory, and these peaks might be the main effective components of UA-lowering (Table 2). Interestingly, peak 11 significantly influenced UA levels although its peak area (content) was small. Conversely, peak 3, 4 and 12 elicited a slight influence on UA level although their peak areas (contents) were large. Samples 2, 9 and 10 were the top three in peak areas of peaks 2 and 5, and coincidentally, they were also the top three in UA-lowering activities. The results indicated that peaks 2 and 5 exhibited a conspicuous effect on the UA-lowering activities.

**Table 2:** The correlation coefficient between UA-lowering and common characteristic peaks.

Peak NO.	Correlation Coefficient	Peak NO.	Correlation Coefficient
1	0.64	8	0.53
2	0.76	9	0.58
3	0.50	10	0.23
4	0.52	11	0.62
5	0.74	12	0.50
6	0.73	13	−0.49
7	0.76	14	−0.33

### 2.4. Results of Chemical Structure Analysis

Peak identification and assignment in HPLC fingerprints of chicory were based on the comparison of their t_R_, HPLC data, MS ion data with reference compounds and previously obtained data [18,19,20]. Table 3 lists the chemical structures of potential effective components.

**Table 3:** Identification of effective components in chicory by HPLC-DAD-ESI-MS^3^.

Peak	t_R_ (min)	M	[M−H]^−^ (*m/z*)	MS-MS (*m/z*)	MS^3^ (*m/z*)	Tentative Identification
1	7.8	340	339.0	176.9	133.0	Aesculin
2	10.9	354	353.1	190.9	172.8, 154.9, 129.0	Chlorogenic acid
5	21.1	474	473.0	310.9	178.9, 148.9	Chicoric acid
6	27.1	516	515.2	353.0	190.9	Isochlorogenic acid A/B/C
7	28.1	516	515.2	353.0, 191.1	179.0, 135.1	Isochlorogenic acid A/B/C
11	42.0	414	412.6	259.0	199.9, 187.0, 171.0, 131.1	13,14-*seco*-stigma5(6),14(15)-diene-3α-ol

The results showed that aesculin, chlorogenic acid, chicoric acid, isochlorogenic acid A/B/C and 13,14-*seco*-stigma5(6),14(15)-diene-3α-ol have a close correlation with UA-lowering activities. However, whether the indicating peaks potentially responsible for the given activity are the published effective components or some new one remains unanswered. We future studies we will investigate the bioactivities of aesculin, chlorogenic acid, chicoric acid, isochlorogenic acid A/B/C and 13,14-*seco*-stigma5(6),14(15)-diene-3α-ol. In addition, these results also provide valuable information for future studies on other effective components of chicory.

## 3. Experimental Section

### 3.1. Instruments

HPLC fingerprints of chicory extracts from ten different sources were obtained using a Shimadzu LC-20A system (Shimadzu, Tokyo, Japan), including a binary solvent delivery pump, a SIL-20A auto sampler manager, a column compartment, together with a SPD-M20A diode array detector (DAD) connected to LC solution software. HPLC-ESI-MS^n^ analysis of samples was carried out with an Agilent 1100 series HPLC (Agilent technologies, Waldbronn, Germany) equipped with a DAD, a quaternary pump and degasser, HP Chemstation Color Spectrum Workstation and an XCT plus electrospray ion trap mass spectrometer with an electrospray ionization (ESI) source.

### 3.2. Reagents and Samples

The ten batches of chicory samples used in this study were purchased or collected from several provinces in China and labeled according to their origins and harvesting time (Table 4), and were authenticated by Professor Yong-Hong Yan (Traditional Chinese Medicine Appraisal Teaching and Research Section of Beijing University of Chinese Medicine). Quails were purchased from a livestock farm in Shunyi, Beijing, China and were raised in cages under standard hygienic condition, given *ad libitum* access to fodder and water. Air-conditioner and venting system were used to keep appropriate temperatures (24 ± 1 °C) and air humidity (45% ± 5%).

**Table 4:** Raw herbs used in this work.

Sample NO	Sources	Origins	Collection Time
S1	Dalian, Liaoning	*Cichorium intybus* L.	August 2013
S2	Haidian, Beijing	Cichorium intybus L.	October 2014
S3	Dengta,Liaoning	*Cichorium intybus* L.	October 2014
S4	Zhaoqing, Heilongjiang	*Cichorium intybus* L.	October 2014
S5	Moyu, Xinjiang	*Cichorium intybus* L.	October 2013
S6	Neimenggu	*Cichorium intybus* L.	October 2011
S7	Pingshan, Hebei	*Cichorium intybus* L.	August 2012
S8	Shouguang, Shandong	*Cichorium intybus* L.	October 2013
S9	Changji, Xinjiang	*Cichorium intybus* L.	August 2014
S10	Changji, Xinjiang	*Cichorium intybus* L.	October 2014

HPLC grade methanol (MeOH) was purchased from Fisher Chemicals (Pittsburg, PA, USA); analytical grade formic acid was purchased from Beijing Chemical Factory (Beijing, China); a Milli-Q water purification system was used to purify water (Millipore, Bedford, MA, USA). Authentic reference standards of chicoric acid and aesculin were purchased from the National Institute for the Control of Pharmaceutical and Biological Products in China, chlorogenic acid was purchased from Sigma (USA). Yeast extract powder (Basingstoke, Hampshire, UK) was used to build the hyperuricemia quail model (15 g·kg^−1^ once a day) [21]. UA reagent kit was purchased from Biosino Bio-Technology and Science Inc. (Beijing, China; Product No. 141161). Benzbromarone was purchased from Excella GmbH (Feucht, Germany; Product No. 1208248).

### 3.3. HPLC Fingerprints

#### 3.3.1. HPLC Conditions

The samples were injected into the HPLC system. Chromatography was carried out on an Agilent ZORBAX SB-C18 column (4.6 mm × 250 mm, 5 μm), operated at 30 °C The mobile phase was composed of 0.1% formic acid water solution (A) and MeOH (B) system with a gradient elution: 0–2 min, 23% B; 2–15 min, 23%–33% B; 15–20 min, 33%–40% B; 20–27 min, 40%–42% B; 27–40 min, 42%–60% B; 40–50 min, 60%–70% B; 50–51 min, 70%–78% B; 51–60 min, 78%–85% B; and 60–70 min, 85%–90% B. The detection wavelength was set at 254 nm and the sample injection volume was 10 μL. Under the present chromatographic conditions, higher resolution could be achieved for most of the peaks.

#### 3.3.2. HPLC-ESI-MS Conditions

The above HPLC system was interfaced with an Agilent 1100 LC/MSD Trap XCT ESI (Agilent Technologies, Waldbronn, Germany). The HPLC–MS analysis was performed under the same gradient program as HPLC-DAD using 0.1% (v/v) formic acid water solution (A) and MeOH (B). The ESI–MS spectra were acquired in negative mode and using the full scan mode from m/z 100 to 1000. Capillary voltage was 3.5 kV. Drying gas temperature was set at 350°C with a flow rate of 11.0 L/min and nebulizing pressure was of 35.0 psi. Data was processed by HPLC/MSD Trap v. 4.2 software and Data Analysis 2.2.

#### 3.3.3. Preparation of Reference Standard Solution

Standard solutions were prepared by adding accurately weighed amounts of chicoric acid, chlorogenic acid and aesculin to a volumetric flask and dissolving with MeOH (50 mL) to give final concentrations of 51.2 μg/mL, 39.0 μg/mL and 44.6 μg/mL respectively.

#### 3.3.4. Preparation of Sample Solution

Chicory was crushed into powder, and 250 g of the powder was accurately weighed and extracted with 2.5 L of water by heating to reflux for 1 h. The procedure was repeated twice. After extraction, the solution was filtered, then concentrated by a rotary evaporator, and diluted to 600 mL with purified water. Aliquots (2 mL) were taken and then filtered through a 0.45 μm micropore film to yield the sample solutions for HPLC analysis. The procedure was repeated in triplicate. The preparation of all 10 samples was performed in the same way.

### 3.4. Analysis of HPLC Fingerprints

#### 3.4.1. Validation of the Methodology

According to the established method programs, method precision and repeatability were evaluated by analyzing five replicate injections of sample 1 and injections of five samples prepared independently from sample 1 (Table 1), respectively. The stability study of sample 1 was performed at different intervals over 24 h (0, 2, 4, 8, 16, and 24 h).

#### 3.4.2. Similarity of HPLC Analysis

Devised by the Chinese Pharmacopoeia Committee, the HPLC similarity evaluation system (Version 2004A) automatically matched HPLC fingerprints for chromatographic fingerprints of traditional Chinese medicines by a ‘similarity evaluation’ system, and then formed the reference atlas using the median method from the general comparison of the chromatograms of ten batches of chicory extracts. Similarity values between the reference fingerprint and the chromatograms of chicory extracts were calculated by the software.

#### 3.4.3. Hierarchical Clustering Analysis (HCA)

HCA is a multivariate analysis technique which is used to sort samples into groups. In this study, the HCA of sample 1–10 was performed using SPSS statistics software (SPSS for Windows 17.0, SPSS Inc., Chicago, IL, USA). The Average Linkage method was applied as the amalgamation rule and the squared Euclidean distance as metric was used to establish clusters.

### 3.5. UA-Lowering Animal Experiment

#### 3.5.1. Animal Experiment Procedure

A total of 130 quails (38 days of age) were randomly distributed to 13 groups, including control group, model group, therapeutic groups, and positive group, with 10 birds in each group. Quails in the model group were given yeast extract powder diet and received no drug treatment; quails in the control group were given a regular diet and received no drug treatment; quails in the 10 therapeutic groups were given yeast extract powder diet and received 10 batches of chicory 5 g kg^−1^ once a day as treatment, respectively; quails in the positive group were given yeast extract powder diet and received benzbromarone 20 mg kg^−1^ once a day as treatment. All quails were given unlimited access to diet and water, blood was taken once a week after fasting 12 h and they were sacrificed after 3 weeks. The experimental design was approved by the Laboratory Animal Management Committee of Beijing University of Chinese Medicine and conformed to the guidelines of the Institutional Animal Care and Use Committee of China.

#### 3.5.2. Determination of UA

All of the groups were evaluated based on serum UA level, which was measured with a UA kit. The serum UA values from different groups were presented as mean ± standard deviation (SD, *n =* 10) and were evaluated by one-way analysis of variance (ANOVA), where *p <* 0.05 was considered statistically significant.

### 3.6. Spectrum-Effect Relationships Analysis

Canonical correlation analysis is to further evaluate the spectrum-effect relationships between the values of peak areas in HPLC fingerprints and the values of UA from ten therapeutic groups by the SPSS statistical software (SPSS for Windows 17.0).

## 4. Conclusions

In the study, HPLC fingerprints and UA-lowering activities were first combined to research the spectrum-effect relationships of chicory, making it possible to evaluate the internal quality and the potential effective compounds in chicory. The UA-lowering activities of chicory samples were closely related to the main effective compounds, whose levels were affected by the natural conditions of the producing regions. The results indicated that aesculin, chlorogenic acid, chicoric acid, isochlorogenic acid A/B/C and 13,14-*seco*-stigma5(6),14(15)-diene-3α-ol might be responsible for the given activities. The results from the study can also be used in evaluating the quality of chicory as well as providing a theoretical foundation for further study on the biological activity of chicory. More importantly, research on the relationships between HPLC fingerprints and pharmacodynamics of herbal medicines makes the holistic evaluation of the internal quality possible. It also provides a rational approach for discovering the potential effective components from complex mixtures. Above all, the study of spectrum-effect relationships provides a powerful tool for the quality control of herbal medicines.
